# The Association Between Family Social Capital and Female Entrepreneurship

**DOI:** 10.11621/pir.2022.0301

**Published:** 2022-09-15

**Authors:** Anastasia A. Raevskaya, Alexander N. Tatarko

**Affiliations:** a Higher School of Economics, Moscow, Russia

**Keywords:** Entrepreneurial intention (EI), family social capital (FSC), female entrepreneurship, perceived behavioral control (PBC), theory of planned behavior (TPB)

## Abstract

**Background:**

Following the new line of research on Family Social Capital, this work focused on the adaptation and application of the Family Social Capital questionnaire to studying the association between Family Social Capital (FSC) and the intention of Russian females to start a business.

**Objective:**

This study investigated the relationship between three dimensions of Family Social Capital (Structural, Cognitive, and Bonding) and components of Entrepreneurial Intention (EI) operationalized via Ajzen’s Theory of Planned Behavior among females in Russia.

**Design:**

Online survey participants (N=222) were assessed with 1) an adapted version of the FSC questionnaire (Álvarez et al., 2019); and 2) the EI questionnaire previously verified on a large Russian sample within Social Capital research (Tatarko & Schmidt, 2015).

**Results:**

The study confirmed the positive relationship of EI with two dimensions of FSC: Structural FSC (namely, the frequency of time spent with significant family members) and Bonding FSC (namely, family resources that can be activated in various life situations). Both positive relationships are mediated by Perceived Behavioral Control (PBC) — one’s feeling of being capable to act upon one’s intentions. The third dimension of FSC — Cognitive FSC (namely, family cohesion and intra-family trust) — showed no association with the intention to start a business among Russian females.

**Conclusion:**

Russian women with higher levels of EI demonstrated higher investment in family time with significant family members (Structural FSC) and reported exposure to larger intra-family resources (Bonding FSC). These two factors, even though not strengthened with a supportive and trustworthy family atmosphere (Cognitive FSC), provided the sense of confidence and control, which empowered the women with the courage to take preliminary actions with the intention of starting their own businesses.

## Introduction

### Female Entrepreneurship

Entrepreneurship is a major driver of an economy (Carree & Thurik, 2010). A high potential for driving up entrepreneurship both in Russia and worldwide lies within the current gender breakdown among entrepreneurs: only 31% of business owners in Russia are female (Mastercard, 2020). Compared to men, potential female entrepreneurs are more sensitive about their competence and experience (Global Entrepreneurship Monitor, 2021), and “relational capital” becomes an important source for them at the start-up stage and beyond (Dal Mas & Paoloni, 2019). This is particularly the case in collectivistic societies where women employ the power of family and close circles for their entrepreneurial efforts (Katz & Williams, 1997; Yetim, 2008).

A new stream of research that allows for in-depth exploration of these strong, “bonding” ties is Family Social Capital: “social capital developed among family members” (Arregle et al., p.76). However, this recently developed construct has not yet been explored in gender-related contexts, nor in relation to Entrepreneurial Intention.

### Entrepreneurial Intention

Intention is a conscious state of mind that directs attention towards a specific object or pathway of achievement (Bird, 1989). Consequently, behavior is best predicted by an intention, despite complications or time delays in its realization (Kautonen et al., 2015; Krueger et al., 2000).

One of the most influential lines of research on Entrepreneurial Intention (EI) is Icek Ajzen’s Theory of Planned Behavior (TPB), which approaches EI as a non-spontaneous, cognitive decision, or behavioral intention, driven by three forces: behavioral attitude, subjective norms, and perceived behavioral control (Ajzen, 2002; Fishbein & Ajzen, 2010).

Behavioral attitude reflects the person’s perception of the desirability of either performing or withholding a behavior or, in the entrepreneurial context, “the degree to which the individual holds a positive or negative personal valuation about being an entrepreneur” (Liñan & Chen, 2009, p. 596). The TPB implies that a more favorable, positive attitude signifies a stronger intention towards launching a business. This has been confirmed by an extensive meta-analysis (Schlaegel & Koenig, 2014) and a more recent specific meta-analysis on social entrepreneurship (Zaremohzzabieh et al., 2019).

Subjective norms (SN) constitute a contextual social situation, *i.e.,* approval of one’s peer group and family. Subjective norms represent the extent to which an individual perceives his or her behavior as correlating with the norms of his/her reference groups. A specific study on subjective norms, covering multiple ways to measure them and cross-cultural contexts (Heuer & Liñán, 2013) has confirmed that subjective norms have a positive effect on EI.

Perceived behavioral control (PBC) refers to the perceived ease or difficulty of action. Out of the three components constituting EI, PBC has demonstrated the strongest and the most stable positive relationship with EI across different research (Schlaegel & Koenig, 2014; Steinmetz et al., 2021).

**Figure 1.**
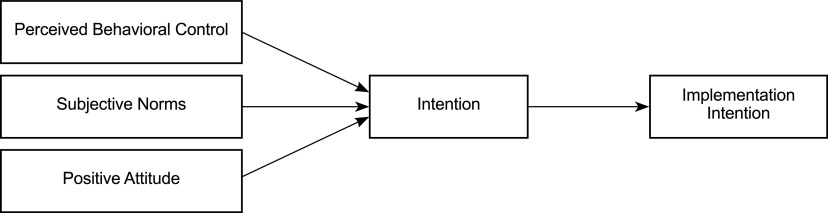
Extended Entrepreneurial Intention model of the TPB (Ajzen, 2002; Gollwitzer, 1999)

According to the TPB, the three above-mentioned components form the grounds for “behavioral intention,” which, in the context of this work, will be further referred to as Entrepreneurial Intention (EI). However, as intention only signifies the direction of action without measuring any actions in the direction of the chosen activity, one more component — “Implementation Intention” — has been proposed to measure the actual steps that an individual is ready to undertake to realize the intention (Gollwitzer, 1999). The extended TPB model including Implementation Intention is shown in *Figure 1* (Ajzen, 2002; Gollwitzer, 1999).

### Social Capital as Antecedent of Entrepreneurial Intention

Intentions in general, and EI as such, are powerful predictors of behavior, as they require prolonged cognitive activity (analyzing, planning, etc.) and thus are more likely to occur under favorable conditions of one’s social structure: availability of role-models, peer-group encouragement, and support, etc. (Krueger et al., 2000). A supportive social environment within the nearest community of family and friends is strongly associated with the probability one would launch a business (Davidsson & Honig, 2003).

Social Capital (SC) is a unifying concept, encompassing the complexity of one’s social relations, thus serving as a reliable antecedent of EI (Liñan & Santos, 2007). By definition, SC is a “stock of social good will… upon which individuals may draw in their efforts to achieve collective or personal objectives” (Furstenberg & Kaplan, 2004, p. 221).

A study based on World Values Survey data discovered that social capital significantly correlates with the self-employment rate, an equivalent of entrepreneurship (Doh & Zolnik, 2011). Research on individual SC has also demonstrated that it acts as a facilitating force to start a new business (Fouratti & Afes, 2011). Recent research in Russia revealed that an intention to start a business correlates with higher individual social capital (Tatarko & Schmidt, 2016).

### Family Social Capital

SC is a complicated concept which can be measured on different levels (societal, group, and individual); however, the shift of research focus to the family and the level of one’s closest networks is a relatively recent development. There is still lack of agreement on a single definition of Family Social Capital (FSC).

The line of research inherited from Coleman (1988) defines FSC as bonds solely between parents and children, the sum of parental attention and time invested into children’s activities and well-being (Hoffmann & Dufur, 2018). However, in Pierre Bourdieu’s “The Forms of Capital” (1986), family is treated as a “united body” with a “solidarity of interests.” Bourdieu’s “network” concept invites a broader understanding of family and definition of FSC as social capital developed among family members (Arregle et al., 2007). Russian scholars define FSC as an intra-family socio-psychological resource which, albeit prone to fluctuations, is foundational for family well-being (Dubrov, 2019). This range of interpretations posits the question of defining the family itself.

Family or “family systems” are undergoing rapid change due to the development of medical technology, increasing social stratification, and cultural changes (Furstenberg, 2019).

A nuclear family — married adults with their offspring — has become too narrow a concept as it excludes elderly relatives, in-laws, and other extended family members whose social “stock” adds up to the social capital of the family in Bourdieu’s understanding. Thus, the focus should be shift ed towards “significant” family members, or those whom a person perceives as such. This approach was explored in a series of qualitative studies among families bringing up children with disabilities: participants were asked to spontaneously list all individuals whom they considered family; the resulting lists included distant living relatives and non-related helpers (Widmer et al., 2013).

Thus, as a compromise between the conceptual treatment of family as a nuclear household and any close friendly circle perceived as family, for the purposes of this work, FSC will be defined as social capital developed among family members (Arregle et al., 2007), where “family” will be treated as a subjective construct as perceived by an individual, or “significant family” extending beyond the nuclear family and household, yet limited to blood relatives and in-laws.

### Family Social Capital Operationalized

As FSC is a more recent outgrowth of SC studies, it inherits the earlier conceptual framework describing SC operationalization.

One of the foundational distinctions of SC is its division into Structural and Cognitive (Uphoff , 2000). Cognitive SC is comprised of perceptions people have regarding their networks, such as cohesion and control, trust, and reciprocity, whereas Structural SC refers to the frequency of network communication and its morphology: density, connectivity, and hierarchy (Nahapiet & Ghoshal, 1998).

**Figure 2.**
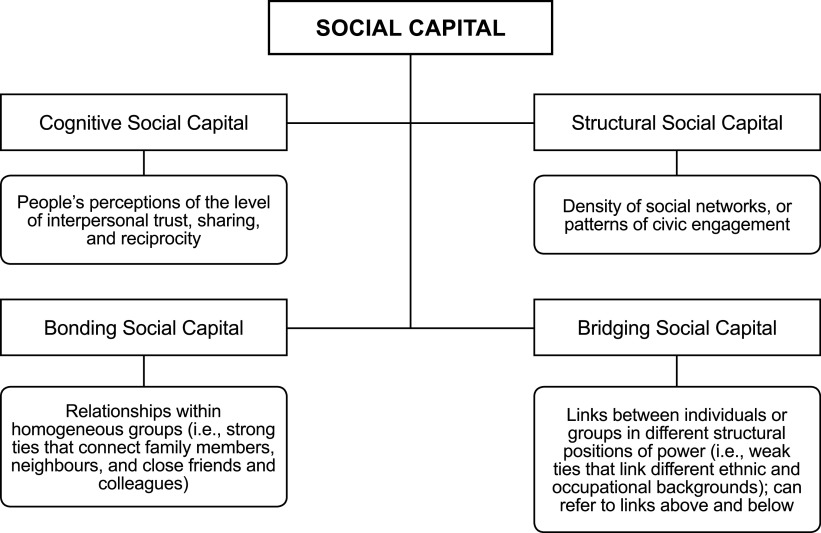
Conceptual schema of Social Capital (Murayama et al., 2012, p.180)

Additionally, SC has been divided into the categories of bonding and bridging (Putnam, 2000). Strong, or bonding, ties originate in the closest community of friends and relatives, whereas weak, or bridging, ties link more distant societal connections with individuals of dissimilar social identity. *Figure 2* depicts a conceptual schema of SC (Islam et al., 2006; Murayama et al., 2012).

This schema became the foundation for a line of research on SC and health, which primarily dealt with adolescents and their well-being; it brought the innovative Family Social Capital (FSC) approach to the field and resulted in the development of the first FSC Questionnaire (FSCQ) (Álvarez et al., 2019) which validated the constructs and their reliability. However, the decision was taken to omit the originally developed sections measuring Bridging and Bonding SC from the final version of the FSCQ, because adolescents, the age group that was selected for the purposes of research, had difficulties identifying the economic and positional circumstances of their family members.

Widely used tools for measuring Bridging and Bonding SC include the Position Generator (Lin, 2001), and the Resource Generator (Van der Gaag & Snijders, 2005). The latter focuses on specific domains of domestic activities — skills, advice, and help that can be gained from close connections. Developed specifically to study individual SC (Van Der Gaag & Webber, 2008), the Resource Generator is a relevant tool to apply to family networks. Thus, to compensate for the excluded section within the original FSCQ, our study used the Resource Generator section to re-introduce the measurement of Bonding SC. Bridging SC was not studied as, by definition, family is one’s closest circle, or strongest tie, whereas Bridging SC, or weak ties, represent distant, non-family related connection. The previously introduced concept of “significant” family members also narrows down the network in question to close ties, or Bonding SC.

### Study Hypotheses

The studies on TPB within the entrepreneurial context have shown that antecedents of EI originate in the wider social environment (Liñan & Santos, 2007; Santos et al, 2016). As family plays a more important role in women’s social networks than men’s (Moore, 1990; Yetim, 2008), this makes FSC a relevant concept to study in relation to the three dimensions of EI specifically for the female population.

The purpose of our study was to investigate the relationship between three dimensions of Family Social Capital (Structural, Cognitive, and Bonding) and components of Entrepreneurial Intention (EI) operationalized via Ajzen’s Theory of Planned Behaviour among females in Russia.

This study first aimed to verify the extended Ajzen’s TPB model. As confirmed by a meta-analysis on TPB applied to EI (Shlaegel & Koenig, 2014), perceived behavioral control, subjective norms, and positive attitude serve as reliable grounds for higher EI. Thus, we formed the following hypotheses:

H1.1. The higher the level of perceived behavioral control in relation to entrepreneurship, the higher is the level of entrepreneurial intention.

H1.2. The higher the level of perceived subjective norms in relation to entrepreneurship, the higher is the level of entrepreneurial intention.

H1.3. The higher the level of positive attitude in relation to entrepreneurship, the higher is the level of entrepreneurial intention.

Also, as suggested within an extended model of EI (Gollwitzer, 1999), a higher implementation intention, or the sum of the actual active steps taken toward opening a business, indicates a higher conversion of intention into behavior. Thus, the following hypothesis was added:

H2. The higher the level of behavioral intention towards entrepreneurship, the higher is the level of implementation intention.

High individual SC has been confirmed to correlate with high perceived behavioral control (Tatarko & Schmidt, 2016). Stronger, more supportive, and more approving social connections of an intender correlate with a positive attitude, subjective norms, and perceived behavioral control in relation to entrepreneurial action (Liñan & Santos, 2007). Bonding SC has been confirmed to correlate with both subjective norms and a positive attitude toward entrepreneurship among students (Vukovic et al., 2017). A new line of research on Family Social Capital, which distinguishes Structural, Cognitive, and Horizontal (Bonding) dimensions (Álvarez et al, 2017), allows us to form the following hypotheses:

H3.1 The higher a woman’s Structural FSC, the higher is her perceived positive attitude, level of subjective norms, and perceived behavioral control as part of her entrepreneurial intention.

H3.2 The higher a woman’s Cognitive FSC, the higher is her perceived positive attitude, level of subjective norms, and perceived behavioral control as part of her entrepreneurial intention.

H3.3 The higher a woman’s Bonding FSC, the higher is her perceived positive attitude, level of subjective norms, and perceived behavioral control as part of her entrepreneurial intention.

**Figure 3.**
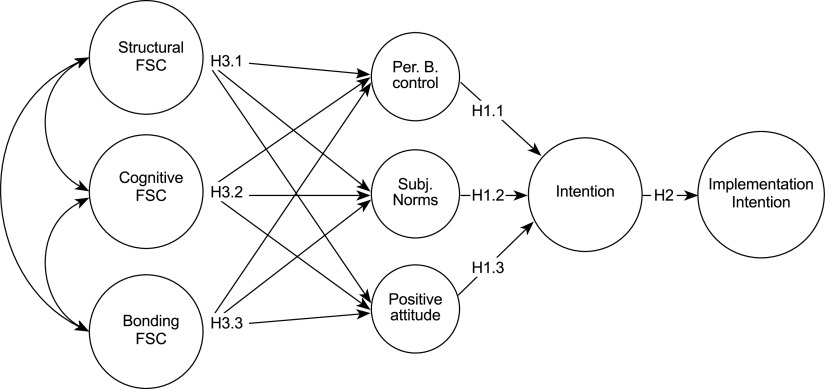
The structural model of the influence of three dimensions of FSC on the Entrepreneurial Intention and Implementation Intention

*Figure 3* represents the conceptual arrangement of the FSC and EI concepts researched within the study.

## Methods

### Participants

The total original sample consisted of 226 females of Russian citizenship, 21 to 70 years of age. Four completed questionnaires of respondents 68 to 70 years of age were excluded as outliers (the women were older than the official Russian retirement rate). Thus, the final sample consisted of 222 women who were 35.6 years of age on average (*SD* = 8.28); most had a higher education (86%); most were currently employed (49%) or unemployed (21%), with only a minor percentage of self-employed / freelance workers (7%); their average incomes ranged within 25000 to 60000 RUB per household member (57%).

### Procedure

The data for the study was collected via an online questionnaire applying the “snowball” technique. The questionnaire was hosted on the 1ka electronic platform (www.1ka.si) and was circulated in social networks, and among public groups of Russian-speaking females devoted to education, as well as general interest students’ communities. The completion time averaged 13 minutes. The subjects were given an overview of the study and provided informed consent before taking the questionnaire. Participation in the study was voluntary with no financial reward.

### Materials

#### Entrepreneurial Intention Questionnaire

The Entrepreneurial Intention Questionnaire was developed and verified based on Ajzen’s TPB questionnaire (Ajzen, 2002) and covered five components of Entrepreneurial Intention: 1) behavioral intention; 2) behavioral attitude; 3) subjective norms; 4) perceived behavioral control; and 5) implementation intention (Tatarko, 2013; Tatarko & Schmidt, 2016). Below is a description of the scales within each dimension.

Behavioral intention (α = .87) was measured using a two-item questionnaire on a six-point Likert scale. Example: “How likely is it that you will start a business within the next two years?”; answers ranged from 1 (*very unlikely*) to 6 (*very likely*).

Positive attitude (α = .90) was measured using two statements. Example: “The idea of starting a business within the next two years is for me . . .”; answers ranged on a six-point Likert scale from 1 (*very inappropriate*) to 6 (*very appropriate*).

Subjective norms (α = .36)^1^ was measured using two items. Example: “Most people who are important to me think I should start my own business within the next two years.” For both statements, answers ranged on a six-point Likert scale from 1 (*strongly disagree*) to 6 (*strongly agree*).

Perceived behavioral control (α = .75) was measured using two items. Example: “For me to start a business within the next two years is . . .”; the answers ranged on a six-point Likert scale from 1 (*very difficult*) to 6 (*very easy*).

Implementation intention (α = .82) was measured using three items. Example: “Are you currently saving money for your intention to start a business?”; the answers ranged on a six- point Likert scale from 1 (*No, I am not*) to 6 (*I have been actively doing this/have already done this*).

#### Family Social Capital Questionnaire

The Family Social Capital Questionnaire was constructed for three dimensions of Social Capital: Structural, Cognitive, and Bonding. Structural and Cognitive Social Capital measurement were based on the recently developed FSCQ (Álvarez, et al., 2019). Structural SC was grouped into four components: communication, shared food, and shared leisure (both within and outside the household). Cognitive SC was grouped into three dimensions: cohesion within the household, cohesion outside the household, and conflicts. Bonding Social Capital was measured with the Resource Generator (Van der Gaag & Snijders, 2005).

### Structural social capital

The communication dimension of Structural FSC (α = .73) was measured using three items. Example: “Frequency of going for a walk with my household family members,” with the answers on a six-point Likert scale ranging from 1 (*never*) to 6 (*twice a week or more often*).

The Shared Food dimension of Structural FSC (α = .81) was measured using three items. Example: “Frequency of preparing food with my household family members,” with answers on a six-point Likert scale ranging from 1 (*never*) to 6 (*twice a week or more often*).

The Shared Leisure (within the household) dimension of Structural FSC (α = .65) was measured using three items. Example: “Frequency of practicing sport with my household family members,” with answers on a six-point Likert scale ranging from 1 (*never*) to 6 (*twice a week or more often*).

The Shared Leisure (outside the household) dimension of Structural FSC (α = .75) was measured using three items. Example: “Frequency of visiting the cinema / museum with my outside-of-household family members,” with answers on a six-point Likert scale ranging from 1 (*never*) to 6 (*twice a week or more often*).

### Cognitive social capital

The Cohesion dimension (within the household) of Cognitive FSC (α = .92) was measured using three items. Example: “We work well as a family,” with answers on a six-point Likert scale ranging from 1 (*never*) to 6 (*all the time*).

The Cohesion dimension (outside the household) of Cognitive FSC (α = .88) was measured using three items. Example: “If there is a problem, we act collectively and cooperate to solve it,” with answers on a six-point Likert scale ranging from 1 (*never*) to 6 (*all the time*).

Conflicts as part of Cognitive FSC (α = .66) was measured using three items. Example: “Frequency of conflict of personal goals within the family,” with answers ranging on a six-point Likert scale from 1 (*never*) to 6 (*all the time*).

### Bonding social capital

Bonding Social Capital (α = .92) was measured using 19 items. Example: “How many significant family members who do not share the household with you, can help you move heavy items while moving to a new location?,” with answers on a six-point Likert-scale ranging from 1 (*never*) to 6 (*five and more*).

## Results

According to the proposed hypotheses, Structural Equation Modelling (SEM) was used to first test Ajzen’s TPB model on the Russian female sample. Consequently three further models assessed each dimension of SC in relation to the TPB model.

### Testing the Extended TPB Model on a Russian Female Sample

**Figure 4.**
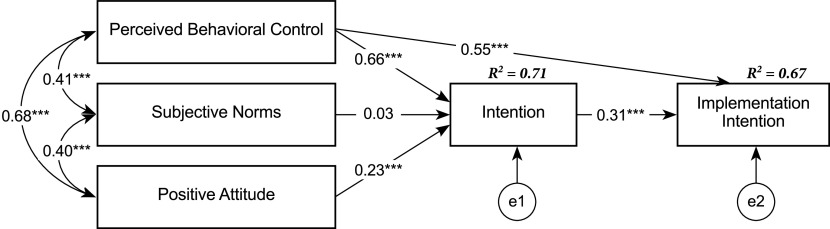
The structural model of the influence of Perceived Behavioral Control, Subjective Norms, and Positive Attitude on Entrepreneurial Intention and Implementation Intention

*Note. Only standardized weights are presented*

Testing the extended TPB model on the Russian female sample (see *Figure 4*) demonstrated good model fit (c2/df = .502; CFI= 1.000; RSMEA = .000; PCLOSE = .590). Perceived Behavioral Control (or feeling in control of one’s entrepreneurial actions) was a major contributor to the intention to open a business (b = .66, p < .001). A higher positive attitude toward entrepreneurship also directly affected behavioral intention, although to lesser extent, but with high statistical significance (b = 0.23, p < 0.001). Subjective Norms was the only component demonstrating no effect on intention. Still, the model explained up to 71% of intention to launch a business and up to 67% of implementation intention, which was addressed while testing the extended TPB model within Hypothesis 2. Implementation Intention (or the actual active steps taken toward opening a business) related positively to both Behavioral Intention (b = .31, p < .001) and PBC (b = .55, p < .001). Th us, out of the three TPB components, it was PBC, or feeling in control of her actions, that provided for a woman’s successful intention to launch a business.

*p < 0.05; **p < 0.01; ***p < 0.001.

### Testing Structural FSC in Relation to TPB

**Figure 5.**
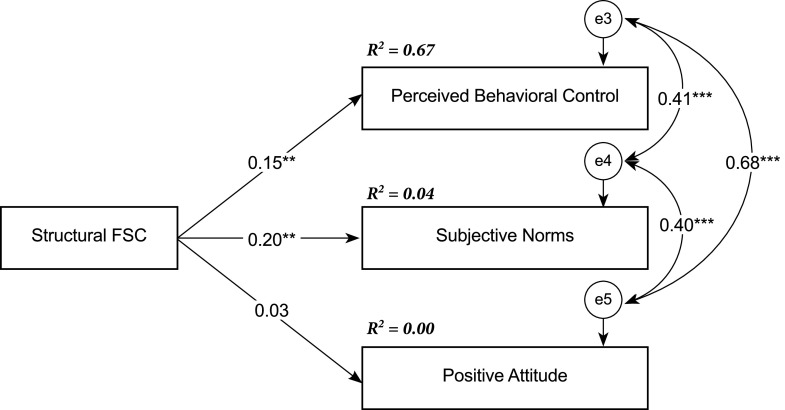
The structural model of the influence Structural Family Social Capital on components of Entrepreneurial Intention

*Note. Only standardized weights are presented*

*Figure 5* demonstrates how the model verified H3.1, our hypothesis that the higher a woman’s Structural FSC, the higher is her perceived positive attitude, level of subjective norms, and perceived behavioral control. The overall model fit was good (c^2^/df = 2.315; CFI= 0.993; RSMEA = 0.076; PCLOSE = 0.203). The structural FSC (or the frequency of various types of activities spent together both within household and with significant family) correlated positively with PBC (b = 0.15, p < 0.01). The same applied to Subjective Norms (b = 0.20, p < 0.05), but there was no direct effect on Positive Attitude. Thus, Structural FSC did have a positive impact on EI through mediation of Perceived Behavioral Control and explained 6% of the dispersion on EI.

*p < 0.05; **p < 0.01; ***p < 0.001.

### Testing Cognitive FSC in Relation to TPB

**Figure 6.**
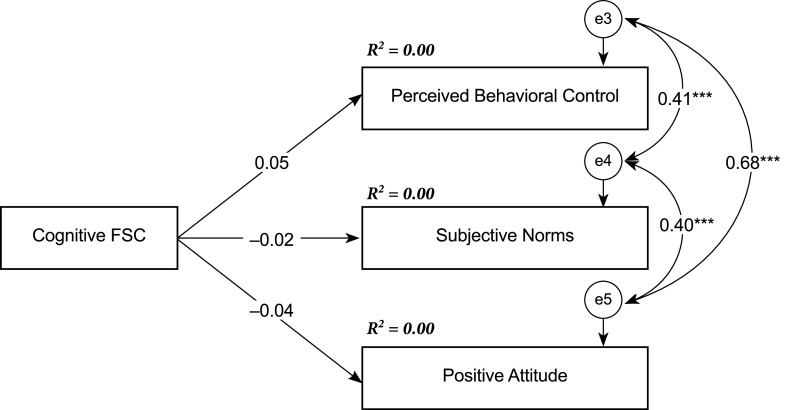
The structural model of the influence Cognitive Family Social Capital on components of Entrepreneurial Intention

*Note. Only standardized weights are presented*

*Figure 6* shows how the model verified H3.2, the hypothesis that the higher the Cognitive FSC, the higher the perceived positive attitude, level of subjective norms, and perceived behavioral control as part of entrepreneurial intention. The overall model fit was good (χ^2^/df = .502; CFI= 1.000; RSMEA = 0.000; PCLOSE = 0.881). Cognitive FSC measures the cohesion and overall quality of relations within the family. This dimension of FSC had no significant impact on any of the components of EI: PBC (β = 0.05, p > 0.05), SN (β = –0.02, p > .05), PA (β = –0.04, p > .05). These results suggest that mutual understanding and a friendly family atmosphere do not impact entrepreneurial intention for Russian women.

*p < 0.05; **p < 0.01; ***p < 0.001.

### Testing Bonding FSC in Relation to TPB

**Figure 7.**
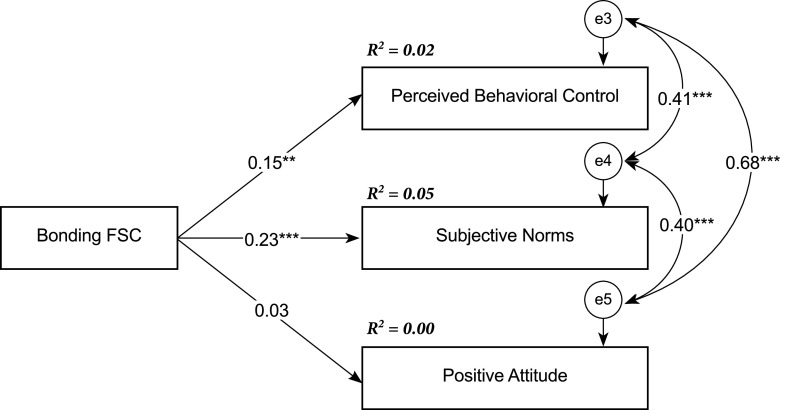
The structural model of the influence of Bonding Family Social Capital on components of Entrepreneurial Intention

*Note. Only standardized weights are presented*

*Figure 7* demonstrates how the model verified H3.3, the hypothesis that the higher the Bonding dimension of FSC, the higher a woman’s perceived positive attitude, level of subjective norms, and perceived behavioral control. The overall model fit was good (χ^2^/df = 0.485; CFI= 1.000; RSMEA = 0.000; PCLOSE = 0.888). Bon ding FSC is the sum of resources that one can mobilize in difficult life situations. It correlates positively with PBC (β = 0.15, p = 0.02). The same applied to Subjective Norms (β = 0.23, p < 0.001), with no effect on Positive Attitude. The findings indicate that a larger, more resourceful family network generally approving of entrepreneurship created conditions for the women feeling in control and starting their own business projects.

*p < 0.05; **p < 0.01; ***p < 0.001.

## Discussion

The hypotheses of this research were partially confirmed.

Higher levels of both Perceived Behavioral Control (H1.1) and Positive Attitude (H1.3) in relation to entrepreneurship signified a higher intention to start a business. This is fully in line with previous meta-analytical research on Ajzen’s TPB model applied to Entrepreneurial Intention (Haus et al, 2013; Steinmetz et al., 2021). Thus, out of all the TPB components, Perceived Behavioral Control, or the perceived ability to be in control of one’s actions, may be the driving force empowering women to start their own businesses, supported by an overall positive perception of entrepreneurial activity. Moreover, this applied to both behavioral intention and implementation intention, as tested within the extended TPB model (H2) and previously confirmed for Russian entrepreneurs (Tatarko & Schmidt, 2016). Women feeling in charge of their abilities were more likely to actualize their intentions, *i.e.,* save money toward launching a business.

On the contrary, hypothesis H1.2 (concerning the positive correlation of Subjective Norms with EI) was not confirmed for this Russian female sample. Subjective Norms refer to overall societal approval of certain behavior. The positive and significant impact of SN has been re-confirmed for an international sample in a meta-analysis on Ajzen’s TPB (Steinmetz et al., 2021). But no such correlation was observed within the current study; interpretation was also limited by low Cronbach’s alpha. However, our findings coincided with those of previous research specific to Russian potential entrepreneurs (Tatarko, 2013; Tatarko & Schmidt, 2016); these non-gender related studies detected no significant effect of Subjective Norms in relation to EI. Recent female-specific research in Pakistan confirmed that perceived national culture had no significant influence on business performance (Shakeel et al., 2020); and as explored in the Spanish environment, a supportive regional entrepreneurial culture did matter for involvement in entrepreneurship, but only for women with a masculine type of gender-role orientation (Liñan et al., 2020).

In Russia this result may possibly be explained by the conflicting attitude toward entrepreneurship, due to the Soviet socialist political paradigm which denounced individual financial profit-seeking. Recent long-term integration into the market economy created a different set of ideals; however, the diversity of attitudes regarding running one’s business, both within different age groups and social classes, does not allow the creation of a pronounced correlation of SN to EI. Thus, the lack of support within her peer group may not play a significant role for a woman considering launching a business.

The last group of hypotheses related to three dimensions of Family Social Capital as predictors of components of EI. These hypotheses were partially confirmed. While the hypothesis related to Cognitive FSC (H3.2) was not confirmed, higher Structural FSC and higher Bonding FSC (tested within H3.1 and H3.3) did predict higher EI.

Structural FSC, or frequency of family time, can take different forms, from joint cooking to video phone-calls. Despite being mostly unrelated to discussing business-related matters, these occasions may provide a sense of foundation and ensure a feeling of stability and being grounded. A Japanese four-year longitudinal study on FSC and self-reported health discovered that more frequent contact with relatives correlated with higher levels of life satisfaction (Jhang, 2019). Thus, higher frequencies of various family interactions resulted in more confidence, more belief in oneself, and more readiness even for such risky actions as entrepreneurial activities.

Bonding FSC is the sum of family resources that one can activate in cases of difficult life circumstances: the number of relatives one can approach to get help. The Resource Generator consisting of 19 items (Van der Gaag & Snijders, 2005) was used to reintroduce the Horizontal dimension, which unites the Bridging and Bonding dimensions of SC, which were not measured within original FSC questionnaire designed for teenagers. Bonding SC significantly impacted Subjective Norms, which in turn positively impacted both EI and II. These findings coincided with those of research on Bonding SC which measured the EI of students in Macedonia and Croatia (Vukovic et al., 2017): Bonding SC positively impacted SN and PBC (for one out of two samples). However, as SN had no direct effect on EI, it was the number of relatives capable of providing assistance and support that impacted EI through generating the feeling of being in control (PBC). Knowing that family will be there for you, even if they have no business experience of their own, creates an important feeling of being supported, and consequently provides grounds for the ability to make the complicated decisions related to running a business.

Interestingly, neither PBC, nor Subjective Norms, nor a Positive Attitude toward entrepreneurship, had any correlation with Cognitive FSC. This dimension concerns family cohesion, the amount of conflict, or ability to work together in difficult situations, shared values, and language; it can be reframed as an overall atmosphere of mutual understanding and shared narratives within the family. The current study revealed that neither a high nor low quality of Cognitive FSC impacted a woman’s intent to pursue her entrepreneurship project. Research on the SC of organizations (Rodrigo-Alarcón, et al., 2018) has emphasized the significance of Cognitive SC for a company’s dynamic capabilities, or continuous entrepreneurial effort. However, shared narratives and mutual understanding within families do not seem to provide any useful background for individual business activities.

Our study focused participants’ attention to their perceived family or significant family members. These relations are often maintained irrespective of positive or negative emotional background. Thus, the desire to launch a business may not be defined by a supportive family atmosphere. Further investigation may explore what women understand as significant family, and whether different types of families, specifically the overall supporting atmosphere within them, influence their entrepreneurial effort.

## Conclusion

The purpose of our research was to verify the recent findings within Family Social Capital theory and adapt the new questionnaire (FSCQ) for the Russian female population in relation to their Entrepreneurial Intention, operationalized as an extended model of Ajzen’s Theory of Planned Behavior.

TPB proved to be a working concept, with components of Perceived Behavioral Control and Positive Attitude predicting EI. The third component, Subjective Norms, appeared insignificant in line with previous research specific to Russia, which suggests that societal approval is not taken into consideration when launching a business, or that other measures need to be adapted to testing Subjective Norms within a female entrepreneurship context. Further investigation is also required due to the diversity of opinions regarding entrepreneurship as a socially approved activity in light of Russia’s Soviet anti-capitalist heritage.

The extended TPB model’s testing of the component of Implementation Intention, or actual active steps like saving for initial investment, also proved to be sustainable for this Russian sample, once again emphasizing the importance of Perceived Behavioral Control. This important feeling of being in charge of one’s ability to perform an action is closely related to self-efficacy and can be called the most significant contributor to a woman’s entrepreneurial intention.

Our findings confirmed that to a small but significant extent, Family Social Capital, especially its Structural and Bonding dimensions, provide the foundations for higher Perceived Behavioral Control. More time spent with significant family members both within and outside the household (Structural FSC), and more connections with family members who can provide help in difficult circumstances (Bonding FSC), served as foundations of PBC and consequently ensured confidence for entrepreneurial intention. Interestingly, shared narratives and overall trust (Cognitive FSC) played no significant role in impacting entrepreneurial action, suggesting that relations with significant family members have a diverse emotional background. Deeper exploration of what constitutes significant family may shed light on whether its morphology may influence Cognitive SC.

To conclude, exploration of Family Social Capital is a promising step for female entrepreneurship research. As the world develops in the direction of appreciating individuality and unique personal expertise (which entrepreneurship represents at its best), it is important to remember that these qualities are rooted elsewhere. Family time and family connections may seem insignificant or even counter-productive when developing one’s individuality. However, investing time in connections with a wider circle of relatives brings valuable benefits through building one’s confidence and sense of grounding and, consequently, the ability to make bold individual moves like opening one’s own business.

## Limitations

The limitations of this study include sampling bias (snowball technique and sample size), which threaten its validity and the ability to extrapolate the results for the general population. The study was correlational with no field or experimental design. Data analysis yielded low reliability and validity within the Subjective Norms component, which may require adoption of different methods to measure this component in future research. Another limitation is that the questionnaire (FSCQ) was developed specifically for adolescents. Thus, the questions were worded and targeted for that specific age group. The adaptation we made for this research was only the minimum necessary; a more extensive approach would require further changes. Thus, this research can be considered a preliminary step toward a more comprehensive adaptation of the questionnaire for adults.

## Footnotes

1 Low Cronbach’s alpha will be addressed in Discussion section as research limitation
